# Functional MRI in optic neuritis: insights into cortical modulation and possible recovery mechanisms

**DOI:** 10.3389/fneur.2025.1675926

**Published:** 2025-12-03

**Authors:** Ayelet McKyton, Ruth Abulafia, Netta Levin

**Affiliations:** fMRI Unit, Department of Neurology, Hadassah Medical Organization and Faculty of Medicine, The Hebrew University of Jerusalem, Jerusalem, Israel

**Keywords:** optic neuritis (ON), multiple scleorsis (MS), functional MRI (fMRI), cortical plasticity, resting state, block desings, connectivity

## Abstract

Optic neuritis (ON) is an inflammatory, demyelinating optic neuropathy commonly associated with multiple sclerosis. It typically presents as monocular visual loss, with most visual functions recovering within several weeks. In addition to spontaneous remyelination, brain adaptation is thought to contribute to the recovery process. In this review, we discuss the role of functional MRI (fMRI) as a powerful tool for examining the cortical changes associated with ON. We explore studies that utilize a range of fMRI methodologies, highlighting their findings and implications for understanding cortical adaptation and recovery following peripheral visual loss. The review begins with traditional block-design fMRI protocols which assess activation strength in response to visual stimuli. It then shifts to analytical approaches that examine resting-state connectivity within the visual system. Advanced techniques, including population receptive field and connective field analyses, are also discussed, emphasizing their ability to probe neuronal spatial properties and detect changes following ON. Finally, we consider emerging fMRI methods that capture the temporal dynamics of cortical activity, underscoring their relevance for elucidating the time-dependent processes underlying cortical adaptation after ON.

## Introduction

Magnetic resonance imaging (MRI), first introduced in the late 1970s, differentiates between tissues by detecting the behavior of hydrogen atoms in a magnetic field. This allows production of high-resolution anatomical images of the brain without the use of ionizing radiation or radioactive tracers. Functional MRI (fMRI) was developed about a decade later in 1990, after Ogawa (1) discovered that oxygen-depleted blood (deoxyhemoglobin) is attracted to a magnetic field. In brief, fMRI measures brain activity by detecting changes in blood flow, specifically the differences between oxygenated and deoxygenated blood, which is expressed as the Blood oxygenation level dependent (BOLD) signal. Importantly, fMRI does not directly measure neuronal activity but rather captures changes in blood oxygenation as an indirect marker of neural function. This is based on the principle that cerebral blood flow and neuronal activation are tightly coupled: when a brain region becomes active, blood flow to that area increases. To assess these dynamics, fMRI scans are conducted over several minutes while participants either rest or perform a task, such as viewing a visual stimulus. The collected data are then analyzed using various models to infer patterns of neuronal activity across the scan.

Over the years, fMRI has become a dominant tool for studying human brain function due to its safety and relative ease of use. Specifically, it has played a key role in advancing our understanding of the visual cortex, offering a non-invasive means to study the functional organization of visual areas, their connectivity and their adaptability to changes in sensory input. In the context of disease, fMRI provides critical insights into altered cortical activity, compensatory mechanisms and the effects of structural damage on visual processing.

**Figure 1:**
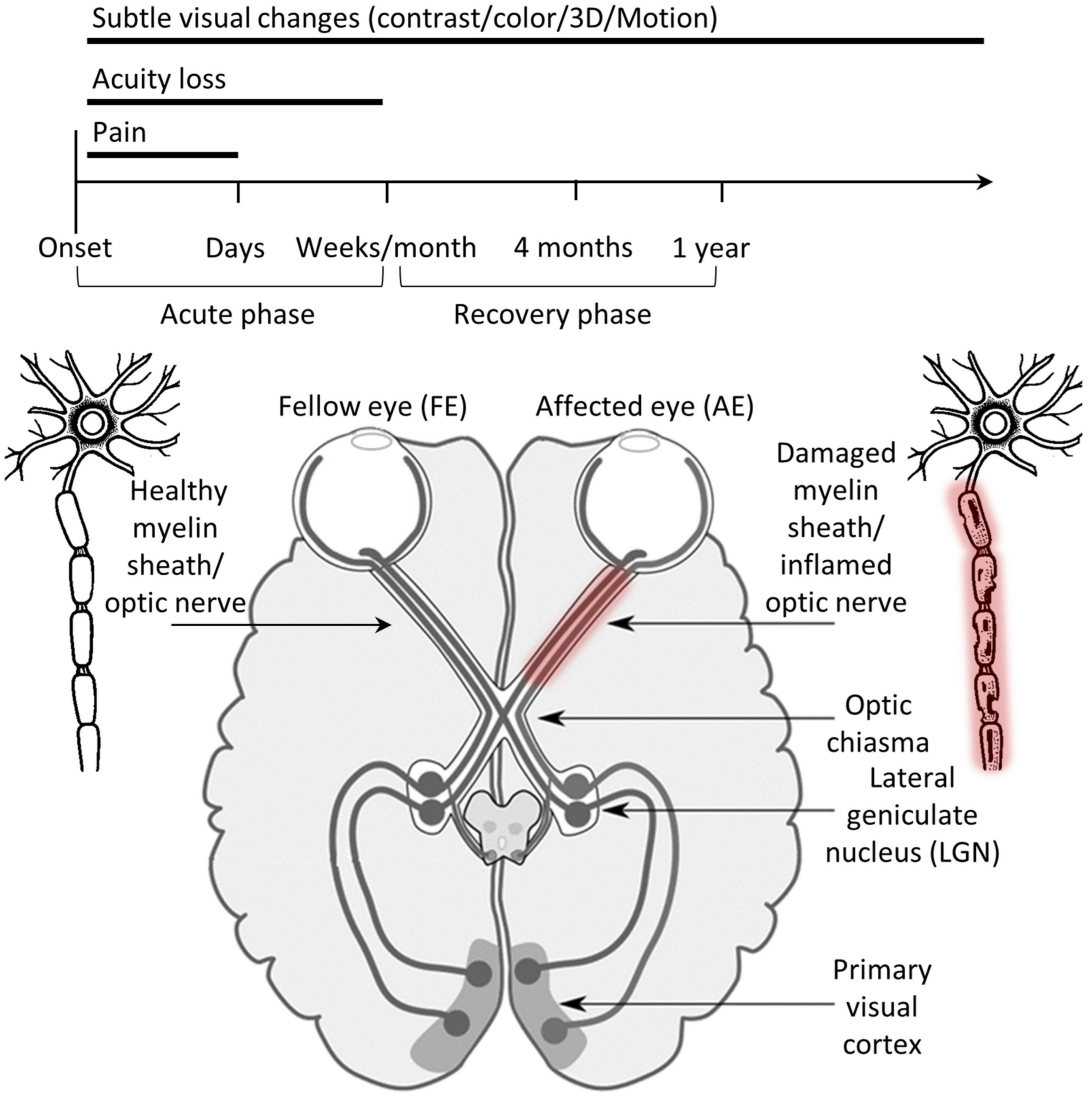
ON in the visual system and its progression. The upper part of the figure shows the different timelines in ON recovery. The lower part shows the human visual system with ON inflammation and damage to a myelin sheath in the optic nerve of the affected eye. Illustration modified from Wikipedia.

Optic neuritis (ON), an inflammatory optic neuropathy, typically presents as acute, transient, unilateral vision loss, often accompanied by pain during eye movement and a temporary reduction in color vision or visual acuity (Figure 1) (2, 3). ON is closely associated with multiple sclerosis (MS), serving as an initial symptom in approximately 15–20% of MS cases and occurring in up to 50% of individuals with MS during their lifetime (4, 5). While ON has historically been linked to MS, the etiological spectrum has expanded with the recognition of neuromyelitis optica spectrum disorder (NMOSD) and myelin oligodendrocyte glycoprotein (MOG) antibody-associated disease (MOGAD) (2). Pathophysiologically, ON results from inflammation-mediated damage to the optic nerve’s myelin sheath, disrupting the conduction of visual signals between the retina and the brain (3). The spontaneous recovery observed in most patients is attributed to a natural remyelination process, facilitated by oligodendrocyte precursor cells that repair the damaged myelin sheath over weeks to months (6). However, some individuals experience residual deficits, such as impairments in visual field coverage, contrast sensitivity and motion perception, which may be due to axonal loss or incomplete remyelination (7). Furthermore, visual evoked potential (VEP) latency delays (prolonged response times of the visual cortex to a stimulus) are observed even in subclinical cases or following previous episodes of ON, persisting despite the resolution of clinical symptoms, which limits the reliability of VEP as a measure of a patient’s functional recovery. Advances in imaging technologies, such as optical coherence tomography (OCT), have further improved the ability to monitor optic nerve damage and recovery, aiding in both diagnosis and prognosis (2).

ON serves as an excellent model for studying cortical adaptation for several reasons. First, as a pre-cortical disorder, it does not directly affect neurons in the cortical visual system, providing a unique opportunity to observe how the visual cortex adapts to degraded input. Second, ON typically presents as a monocular condition, impairing one eye (the affected eye, AE) while sparing the other (the fellow eye, FE), allowing researchers to investigate whether input from the FE is processed differently to compensate for the diminished input from the AE. Third, ON typically includes an acute phase of acuity loss followed by prolonged subtle deficits in contrast, color or motion perception, allowing the study of adaptation processes across different timeframes. Lastly, unlike most visual deficits that primarily affect spatial vision (acuity/field of view), ON also disrupts temporal visual processing. This aspect of vision remains less explored in the context of visual disorders.

Given these factors, numerous studies have sought to identify the neuronal correlates of ON and the mechanisms underlying recovery and adaptation using fMRI. In this review, we present both classical and advanced fMRI analysis techniques, highlighting studies conducted across different ON cohorts. Our goal is twofold: first, to introduce readers unfamiliar with functional imaging to the various fMRI methods available and their appropriate applications; and second, to synthesize findings from these studies to address key questions regarding the disease and its recovery processes.

## ON in block-design visually driven fMRI

**Figure 2:**
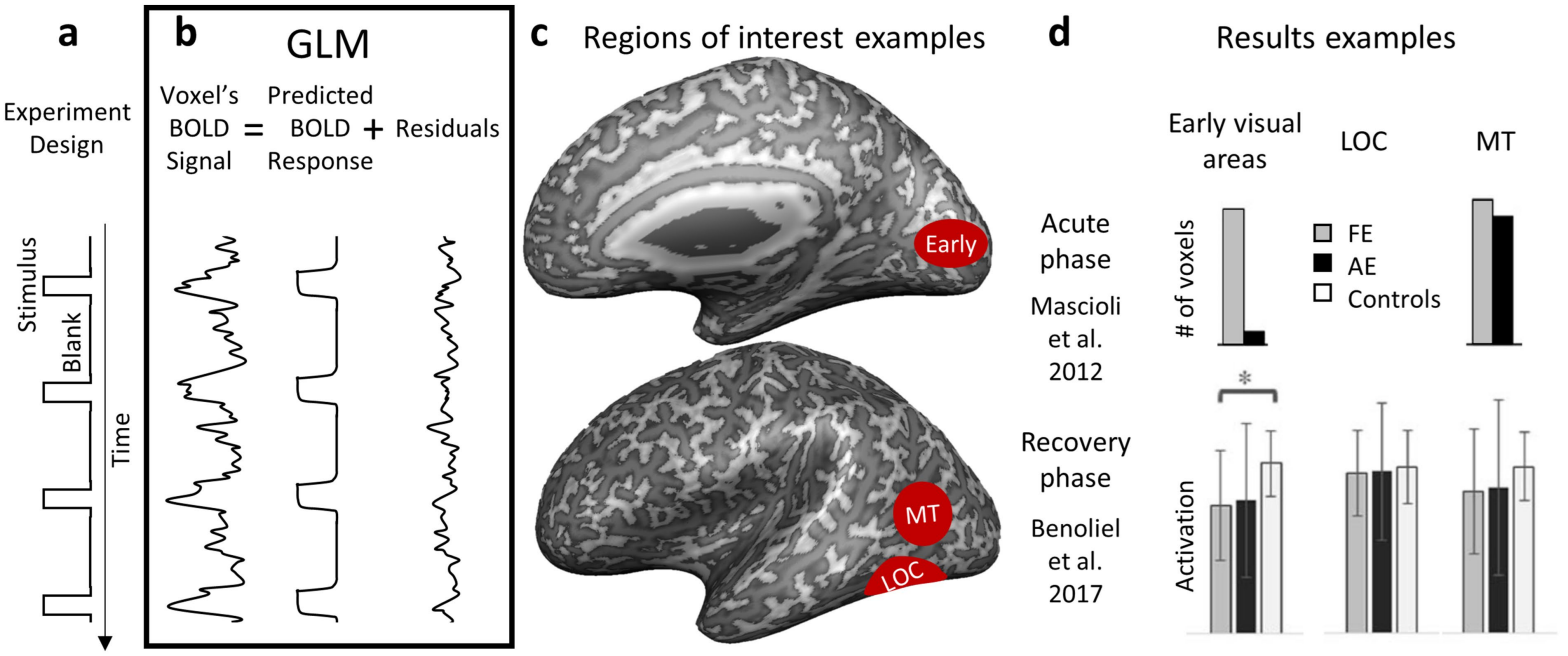
GLM design, analysis and example results. **(a)** Experimental design that alternates between task conditions. In this example, the design alternates between blank and stimulus conditions. **(b)** GLM components. The observed BOLD signal is subtracted from the predicted BOLD response, resulting in the residuals. Statistical tests compare the amount of signal that can and cannot be predicted. **(c)** Schematic examples of regions that can be detected when comparing stimulus vs. blank conditions. **(d)** Result examples showing the number of activated voxels in the acute phase (Patient #1, V1 and MT (8)) or activation strength in the recovery phase (Group data, V2, LOC and MT (9)) within areas of interest.

A block-design visually driven fMRI procedure is a technique widely used in cognitive neuroscience to measure brain activity. This method involves presenting stimuli in structured, time-blocked intervals, enabling researchers to observe changes in brain activity. The experiment alternates between task conditions (e.g., visual stimulus presentation) and baseline conditions (e.g., a blank screen) (Figure 2a). Each condition lasts for a fixed duration and is repeated multiple times throughout the experiment to enhance statistical reliability. Analyses compare the BOLD signal between task and baseline conditions to identify brain regions involved in specific sensory processes. These analyses can also compare task conditions directly between groups or eyes to pinpoint areas with greater activation in one group or eye during stimulus presentation.

To analyze fMRI data from block design experiments, researchers commonly use the general linear model (GLM), a statistical framework that examines how experimental conditions influence the BOLD signal across the brain (10). In this approach, the stimulus presentation schedule is modeled as an independent variable (predictor), while the measured BOLD signal serves as the dependent variable (Figure 2b). The GLM estimates how well the observed BOLD fluctuations correspond to the expected hemodynamic response evoked by stimulus blocks. A key component of this analysis is the hemodynamic response function (HRF), which accounts for the delayed and dispersed nature of the BOLD signal following neural activation (11). By convolving the HRF with the stimulus onset times, researchers generate a predicted BOLD response curve for each condition. Statistical contrasts within the GLM then compare activation levels between task and baseline conditions, revealing brain regions significantly engaged by the experimental manipulation (12) (Figure 2c). Group-level GLM analyses are used to compare activation patterns across participants, groups or conditions (e.g., comparing activation to visual stimuli presented separately to the left vs. to the right eye), providing insights into population-level differences in sensory processing.

When comparing between eyes, a healthy control group is unnecessary if symmetry between eyes is assumed. However, if the goal is to determine whether asymmetry arises from reduced activity in one eye or increased activity in the other, a control group becomes essential. Studies on patients during the acute phase have shown reduced activity in *early visual areas,* such as V1, when stimuli are presented to the affected eye compared to the fellow eye (8, 13–16) (Figure 2d). Some recovery is observed 6–12 months after the acute phase, though inter-eye differences persist (14–16). Comparing activation in these areas during stimulation of the affected eye to controls’ activation levels supports the notion of reduced activity in early visual areas in ON (9, 13, 17) (Figure 2d). This reduced activity is correlated with impaired visual abilities (17, 18) and retinal atrophy (19).

While activity in the early visual cortex is linked to low-level visual properties such as brightness and contrast, activity in *higher visual areas* like the lateral occipital complex (LOC) is associated with stimulus meaning. This selectivity pattern, along with variation in disease severity (20), might explain the inconsistencies in findings on high-level visual cortical activity in ON patients during stimulation of the affected eye. For instance, when meaningless high-contrast stimuli were presented, reduced LOC activity was observed in the affected eye (14). However, this difference was absent when object images were used (16). Notably, ON patients exhibit deficits in contrast perception but not in object recognition. Generally, reduced LOC activity during stimulation of the affected eye was not observed in ON patients, probably reflecting their intact object recognition abilities (9, 14–16).

Similar inconsistencies were found in V5/MT. One study using a static checkerboard display reported no deficits during the acute phase (8), while another using moving stimuli found reduced activity in MT even a year following the acute event (16). Furthermore, cortical activity in response to object-from-motion stimuli was found to be correlated with VEP latencies in ON patients (9). Since ON patients show motion perception deficits (21), these inconsistencies can also be understood by viewing cortical activation as a reflection of the perception. In other words, when presenting static stimuli that are easy for patients to perceive, cortical activation appears unaffected. However, presenting motion, known to be more difficult for patients to perceive, reveals a reduction in fMRI activity.

In summary, results from block-design visually driven fMRI studies reflect the perceptual deficits experienced by ON patients. Low-level visual stimuli elicit reduced cortical activity, particularly in early visual areas, which partially recovers on a similar timeline to low-level visual deficits. Static high-level visual stimuli, which ON patients perceive normally, do not cause reduced cortical activity in higher cortical areas when viewed with the affected eye. Conversely, moving stimuli, known to elicit impaired detection (21) and object-from-motion recognition (16) in ON patients, result in decreased cortical activity.

However, one should remember that while block-design visually driven fMRI offers strong statistical power, it also has notable drawbacks. Prolonged blocks may induce habituation, fatigue or attentional drift (22, 23), and reliance on a canonical hemodynamic response ignores inter-individual and regional variability (24). These factors are especially relevant in optic neuritis, where vascular and neural responses may deviate from healthy norms.

## ON in resting-state fMRI of the visual system

Resting-state functional magnetic resonance imaging (rs-fMRI) is a neuroimaging technique that measures spontaneous fluctuations in the BOLD signal while a subject is not engaged in any specific task (usually with eyes closed or fixating on a cross). These fluctuations are believed to reflect intrinsic neural activity and functional connectivity between different brain regions (25). Unlike task-based fMRI, which examines brain activation in response to specific stimuli, rs-fMRI allows researchers to study the brain’s baseline functional architecture, making it an essential tool for investigating large-scale neural networks.

By analyzing patterns of synchronized BOLD signal fluctuations, rs-fMRI can identify functional networks associated with vision, even in the absence of external stimuli, offering insights into how visual areas communicate and process information in both health and disease (26). Moreover, resting-state connectivity patterns have been used to investigate neuroplasticity, revealing changes in visual networks in response to sensory deprivation (e.g., blindness) or neural damage (e.g., stroke or optic neuropathy) (27).

**Figure 3:**
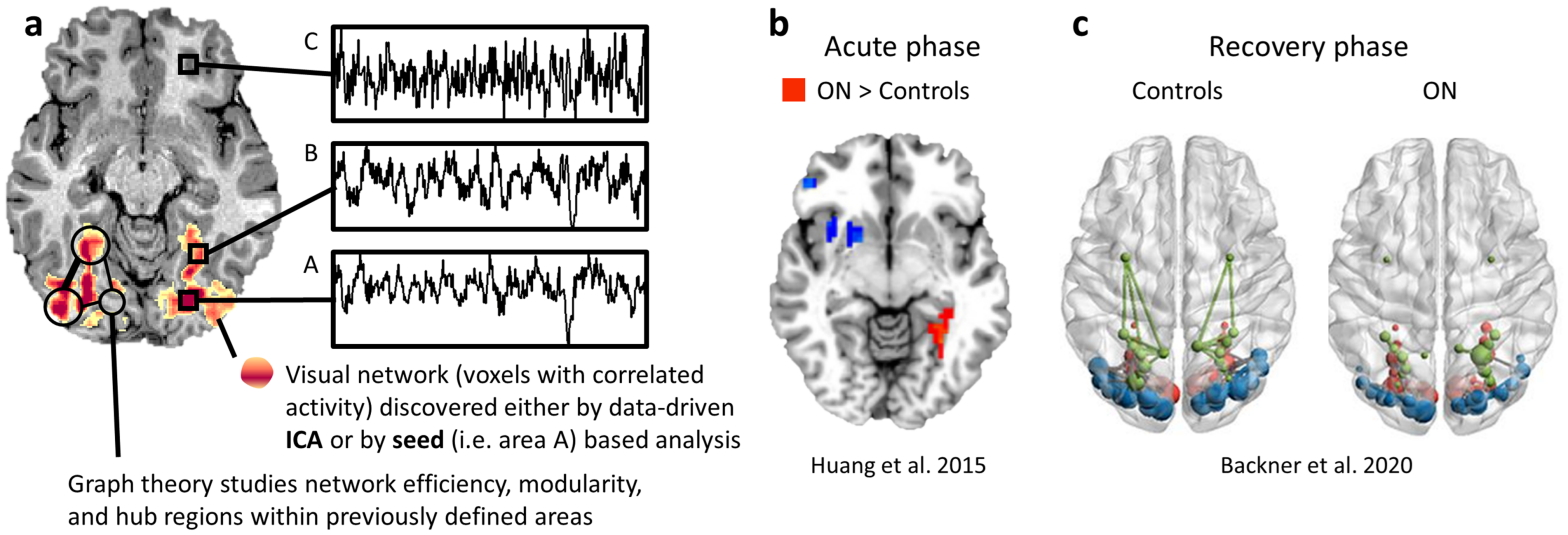
Resting state analyses and results examples. **(a)** The visual network is shown in orange and defined as the voxels in the visual system whose activity is synchronized. Voxels A and B show synchronized activity and are a part of the network, as opposed to voxel C. The network can be defined using ICA or seed-based analyses. Graph theory necessitates pre-defining the different visual areas and testing the efficiency, modularity and hubs within this network. **(b)** Huang et al.’s (28) study shows visual areas with stronger connectivity in acute ON patients than in controls. **(c)** Backner et al.’s (29) study shows stronger connectivity within the visual network in controls than in ON patients. The size of nodes reflects the relative degree of a given region in the network.

To identify functionally connected neural networks, researchers commonly use analytical techniques such as independent component analysis (ICA), seed-based correlation analysis (Figure 3a), regional homogeneity (ReHo), amplitude of low-frequency fluctuations (ALFF) or clustering methods. ICA is a data-driven approach that decomposes rs-fMRI data into statistically independent spatial components, allowing for the identification of distinct resting-state networks, including the visual network (30). This method does not require predefined regions of interest, making it particularly useful for uncovering novel or unexpected connectivity patterns. Seed-based correlation analysis, on the other hand, involves selecting a predefined seed region, such as V1, and calculating the temporal correlation between its BOLD signal fluctuations and those in other brain areas. This approach is useful for examining the functional relationships between specific visual regions and their broader connectivity with attentional, memory, or multisensory integration networks (31). Recent advances in rs-fMRI have also incorporated graph-theoretical approaches to study network topology, enabling researchers to quantify properties such as network efficiency, modularity, and hub regions within the visual system (Figure 3a). Regional homogeneity (ReHo) assesses the similarity or synchronization of the time series of a given voxel with its neighboring voxels, providing information about local functional connectivity. Amplitude of low-frequency fluctuations (ALFF) measures the intensity of spontaneous fluctuations in the BOLD signal, reflecting the level of regional spontaneous neuronal activity. Additionally, clustering methods such as k-means clustering have been employed to identify distinct brain states by grouping similar patterns of functional connectivity. rs-fMRI has been instrumental in revealing altered visual network connectivity in various visual conditions such as ON (32), providing a foundation for understanding the neural basis of visual processing and potential targets for therapeutic intervention.

Several studies have examined rs-functional connectivity patterns following ON at various time points after the acute event, employing diverse analytical approaches and control groups for comparison.

Comparisons of functional connectivity within the visual system in patients with acute ON and in healthy controls, using the amplitude of low-frequency fluctuations (ALFF) technique, unexpectedly revealed enhanced connectivity in the patient group in the occipital and temporal regions of the visual system (28, 33) (Figure 3b).

At later stages, several months after the acute episode, functional connectivity within the visual network was compared between patients with clinically isolated syndrome presenting with ON (CIS-ON), CIS patients with motor or sensory symptoms but without ON (non-ON CIS), and healthy controls. These analyses demonstrated higher connectivity in CIS-ON patients relative to non-ON CIS patients (29, 34), but lower connectivity than in controls (29, 35) (Figure 3c). Importantly, lesion load along the visual pathways was comparable between CIS-ON and non–ON CIS groups in these studies (29, 34) and therefore did not account for the observed differences in connectivity measures.

In the same cohort, diffusion tensor imaging (DTI) revealed reduced diffusivity along the optic tracts of patients with ON, suggesting an extension of axonal injury from the affected optic nerve. However, neither the optic radiations nor the splenial fibers showed evidence of reduced structural integrity. Thus, in this specific study, despite an intact post-geniculate anatomical network, functional connectivity within the visual network was elevated in the ON cohort.

To further investigate the influence of underlying inflammatory disease type on connectivity patterns following ON, Backner et al. (29) applied graph theory-based analyses to compare connectivity in patients with neuromyelitis optica spectrum disorder (NMOSD), as well as in the CIS-ON and non–ON CIS groups. They reported that all patient groups exhibited reduced visual network density (total number of connections) compared with controls; however, ON groups showed a higher connection degree (number of links per region) than the non-ON CIS group. Notably, network efficiency (how easily information is transferred) and modularity (the extent to which the network is divided into subclusters) were reduced in the CIS groups but preserved in NMOSD patients. These findings suggest that cortical visual network alterations depend not only on the occurrence of ON but also on the underlying disease pathology.

Finally, in patients with progressive multiple sclerosis (MS), graph theory-based analyses were used to assess whether functional visual networks remain discernible even years after an acute ON episode. These studies found no significant differences in functional connectivity between ON and non-ON MS patients (36). Although functional metrics did not differ between groups, anatomical global efficiency and network density, measured using DTI, were significantly lower in the ON group, despite comparable lesion loads.

Overall, using resting-state fMRI as a tool to investigate visual system connectivity in ON patients provides a complex picture regarding the disease progression. Two processes seem to run in parallel. First, following the ON episode, connectivity enhancement occurs in the visual cortex, perhaps as an attempt of the brain to adapt to the impaired input, and then declines over the course of months. This process results in high connectivity of ON patients first as compared to controls and later as compared to non-ON multiple sclerosis patients whose visual input is adequate. In parallel, a continuous overall decrease in connectivity over months and years is evident in all multiple sclerosis patients. This results in lower connectivity of ON patients compared to controls. It is possible that the visual system, while initially able to recruit adaptive mechanisms, loses this ability over time as disease progression continues to affect white matter integrity.

Although rs-fMRI provides valuable insights into intrinsic visual network connectivity, it is sensitive to confounds such as head motion, physiological noise and scanner drift, which can mimic or obscure true neural correlations (37, 38). The biological meaning of resting-state fluctuations also remains debated, as connectivity patterns may not directly reflect underlying neuronal communication (39). Moreover, analytic choices such as seed placement or preprocessing strategies can strongly influence results, limiting reproducibility across studies.

## ON in population receptive field

An additional aspect of brain plasticity in the visual system may involve modulation of visual field representation, which refers to reorganization of how visual space is mapped onto cortical areas. Studies have shown that the organization of visual field representations is not rigid; rather, it can adapt over time based on experience and external stimuli. These changes in cortical representation can be noninvasively observed using advanced neuroimaging techniques such as population receptive field (pRF) analysis, which provides insights into how visual information is processed and reorganized in the brain (40). The pRF technique models the portion of the visual field within which visual stimuli can evoke a neural response in a target voxel (a three-dimensional MRI pixel), offering a fine-grained view of neural selectivity and spatial encoding in the visual cortex.

**Figure 4:**
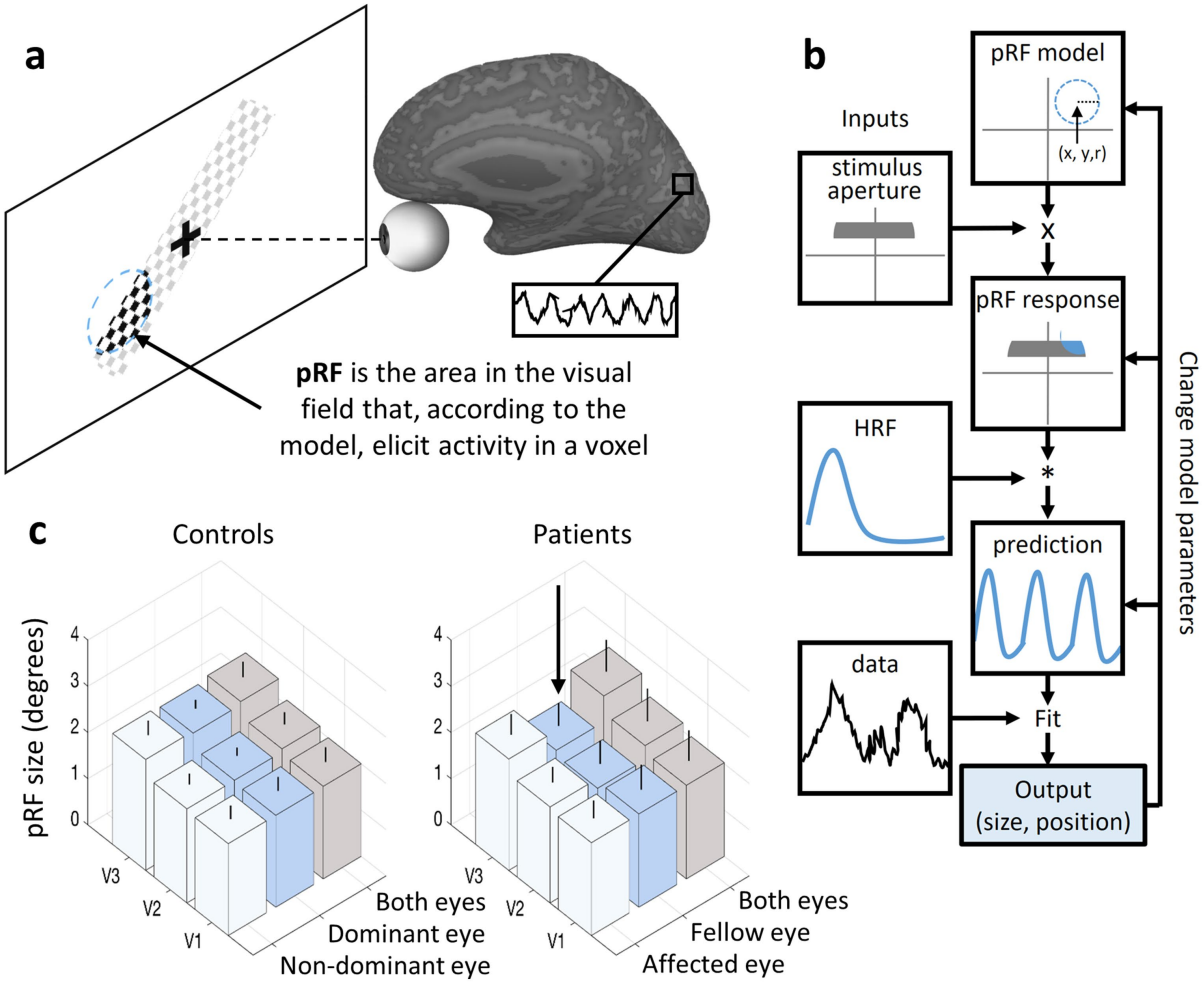
pRF analyses and results example. **(a)** A diagram representing a participant in a pRF experiment presented with a dynamic visual stimulus (drifting checkerboard bar) that evokes neural responses in a visual cortical voxel. A dashed circle represents the pRF model output (size and location in the visual field) for the voxel in the visual cortex marked by a black square. **(b)** The pRF model applied to each voxel in the visual cortex. **(c)** On the left, a typical pRF size pattern in controls shows increases in pRF size along the visual hierarchy (V3 > V2 > V1) regardless of the eye being used. On the right, the pattern of pRF enlargement along the visual hierarchy is not seen in ON patients when the stimulus is presented to the fellow eye (41). Error bars indicate the standard deviation of the mean.

The measurement of pRFs relies on fMRI combined with carefully designed visual stimuli and computational modeling. During a pRF mapping experiment, subjects are presented with dynamic visual stimuli, such as moving bars, expanding rings or rotating wedges, that systematically cover different regions of the visual field (Figure 4a). These stimuli evoke neural responses across the visual cortex, which are recorded using fMRI by measuring BOLD signals. A computational model is then applied to estimate the pRF properties of each voxel in the visual cortex, typically assuming a Gaussian or difference-of-Gaussians function to represent the receptive field’s spatial selectivity (40) (Figure 4b). This model is fitted to the fMRI responses to determine the pRF center location and size. The resulting pRF maps provide a detailed visualization of how the visual field is represented in cortical areas, allowing researchers to investigate systematic changes in response to learning, damage or sensory adaptation.

In healthy subjects, pRF characteristics follow two principal patterns that reflect the functional organization of the visual cortex. First, there is a positive linear relationship between pRF size and eccentricity, where pRF size increases with greater eccentricity, meaning that cortical voxels that process peripheral visual stimuli have larger pRFs than those responding to stimuli near the fovea. Second, the slope of this function increases along the visual hierarchy, meaning that as visual information progresses from primary visual cortex (V1) to higher-order areas (such as V2, V3 and beyond), the receptive fields become larger (Figure 4c; left). This hierarchical pattern enables increasingly abstract and global processing of visual information, facilitating object recognition, motion perception and other complex visual tasks.

Recently, we have identified unique features in the visual field map organization of ON subjects, particularly concerning their fellow eye (41). While the typical increase in pRF size along the eccentricity axis was preserved, the classical pattern of pRF enlargement along the visual hierarchy was disrupted when stimuli were presented to the fellow eye (Figure 4c; right). This disruption was driven by reduced receptive field sizes in the extrastriate cortex, which differed from those of sighted controls and were correlated with prolonged conduction velocities in the affected eye’s optic nerve. We proposed that during the first year following an acute ON event, a spatial adaptation mechanism is mediated by the fellow eye.

Although pRF mapping offers a fine-grained view of visual field organization, it relies on strong assumptions about receptive field shape and stability, typically Gaussian models, that may oversimplify neural tuning (40). The approach is also sensitive to eye movements, fixation instability and attentional fluctuations, which can distort receptive field estimates. Moreover, the spatial resolution of fMRI and the indirect nature of the BOLD signal impose limits on the precision with which individual receptive fields can be inferred.

## ON in connective field

An additional aspect of brain plasticity in the visual system involves modulation of data integration, which refers to the way different visual areas interact and share information to process complex visual inputs. This plasticity plays a critical role in adaptive changes following sensory deprivation, neurological injury or visual training, demonstrating the brain’s remarkable ability to reorganize functional connections. While traditional population receptive field (pRF) analyses focus on how individual voxels in the visual cortex respond to specific regions of the visual field, a complementary approach called connective field (CF) analysis provides insights into how information is integrated between different cortical areas. These changes in connectivity patterns can be noninvasively observed using CF analyses, which extend the concept of pRF mapping by identifying the spatial extent of connectivity between voxels across different cortical regions (42). CF analysis is particularly valuable for understanding how higher-order visual areas receive and process information from earlier visual areas, revealing the hierarchical structure of visual processing.

**Figure 5:**
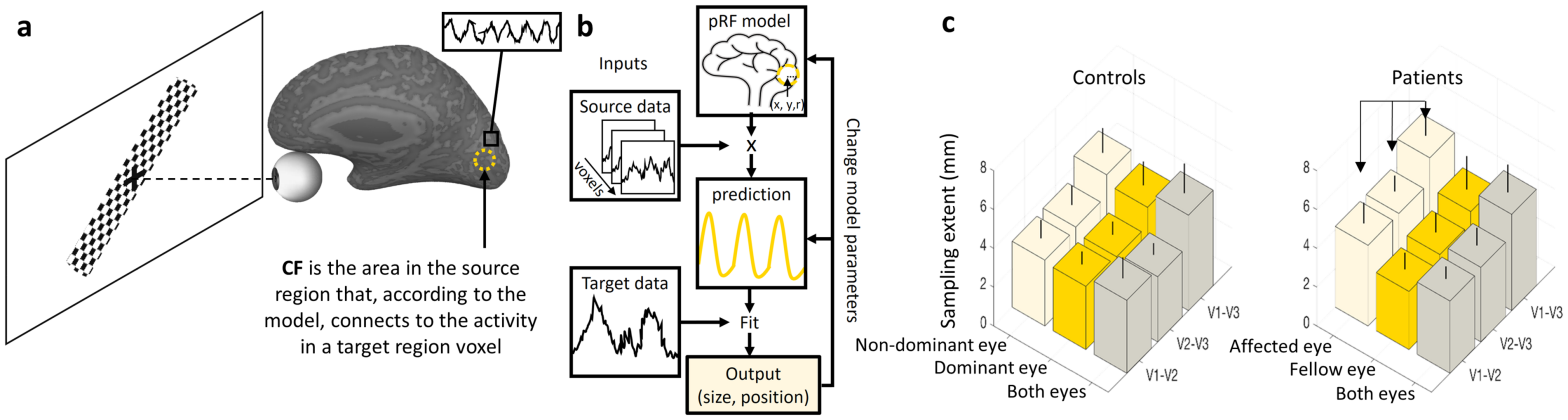
CF analyses and results example. **(a)** A diagram representing a participant in a CF experiment presented with a dynamic visual stimulus (drifting checkerboard bar) that evokes neural responses in the visual cortex. A dashed circle represents the CF model output (size and location in the source area) for a voxel in the target region marked by a black square. **(b)** The CF model applied to each voxel in the target region. **(c)** On the left, a typical CF sampling extent pattern in controls shows an increase in CF size along the visual hierarchy (V1-V3 > V1-V2) regardless of the eye being used. On the right, ON patients show enlargement in CF sampling extent for the affected eye relative to the fellow eye (41). Error bars indicate the standard deviation of the mean.

Connective field (CF) analysis, introduced by Haak et al. (34), models the portion of one cortical region that elicits a response in a target voxel located in another brain region. This approach builds on principles from pRF analysis but shifts the focus from mapping receptive fields in visual space to mapping functional connections between cortical regions. In CF analysis, fMRI data are collected while subjects view dynamic visual stimuli, such as moving bars or expanding rings, which evoke neural responses across the visual cortex (Figure 5a). The CF model then estimates the location and size of the effective input region from a source area (e.g., V1) that drives activity in a target area (e.g., V2 or V3). By applying a Gaussian or difference-of-Gaussians model, similar to pRF estimation, researchers can infer the extent and specificity of connectivity between visual areas (Figure 5b). This method provides a powerful framework for studying large-scale cortical integration, offering insights into how visual information is transferred and processed along the visual hierarchy. Furthermore, CF analysis has been used to explore cortical reorganization in clinical populations, such as individuals with visual field deficits due to retinal or cortical damage, providing a deeper understanding of compensatory mechanisms in the visual system (42, 43).

In healthy subjects, CF analysis reveals characteristic patterns across different visual areas. The output of the analysis, the sampling extent, reflects the size of a source area (A) from which neurons in a target area (B) receive their input. A small sampling extent for a voxel in area (B) indicates that it is connected to a relatively small, focused region in area (A). In contrast, a large sampling extent suggests that the voxel in (B) draws input from a much broader region in (A). Akin to pRF characteristics, CF size increases along the visual hierarchy when the source area is kept constant. For example, when mapping connectivity from V1 to higher areas such as V2, V3 and beyond, the CF size expands, suggesting broader integration of visual information in higher-order areas. This pattern aligns with the general principle of hierarchical visual processing, where early visual areas encode precise spatial details, while later areas integrate larger-scale visual patterns and object features. These findings highlight the role of CF analysis in understanding both normal visual function and adaptive changes due to neural plasticity, making it a crucial tool in vision science research (42, 43).

Recently, we have studied CF patterns in ON (41) (Figure 5c; left). We observed that the known increase in CF size along the visual hierarchy was preserved for both ON and control groups, reflecting the broader integration of visual information in higher-order areas. However, a significant inter-ocular difference was noted in the ON group, with oversampling (larger CF) in the affected eye (Figure 5c; right). These findings suggest a compensatory mechanism in the visual cortex: the larger CF size in the affected eye may represent an adaptive response to impaired visual input caused by ON, likely by increasing the extent of processed data. Similar compensatory mechanisms have been observed in previous CF studies (44–46).

While connective field analysis offers valuable insights into inter-regional visual cortex integration, it relies on assumptions about the shape and linearity of functional connections, which may oversimplify the true complexity of cortical interactions (42). The method is sensitive to noise, head motion, and variability in hemodynamic responses, potentially affecting the precision of inferred connectivity patterns. Additionally, CF estimates are constrained by the spatial resolution of fMRI and the indirect nature of the BOLD signal, limiting the ability to resolve fine-grained, neuron-level connectivity.

## ON in temporal receptive fields

Additional fMRI techniques could also help enrich our understanding of ON. Temporal receptive fields (TRFs), like spatial receptive fields, exhibit a systematic increase in size along the visual hierarchy, reflecting the brain’s capacity to process visual information over progressively longer time scales (47–49). TRFs define the duration over which a neuron or voxel integrates incoming visual stimuli, influencing how dynamic visual information, such as motion, object continuity or complex scene changes, is perceived. In early visual areas such as V1, TRFs are relatively short, responding to rapid changes in visual input with high temporal precision. As processing advances to higher-order areas such as V2, V3 and beyond, TRFs become progressively larger, allowing for the integration of more extended sequences of visual input. Larger temporal integration windows enable higher visual areas to extract meaningful patterns, track motion over time and contribute to perceptual stability.

Temporal receptive field analysis is particularly relevant for understanding visual deficits associated with diseases such as ON that affect temporal processing. Further refining our understanding of TRFs across the visual hierarchy could pave the way for novel interventions targeting temporal processing deficits in clinical populations.

## Discussion

In this review, we examined a range of functional MRI experimental protocols and analytical techniques aimed at improving our understanding of the effects of optic neuritis (ON) on the visual system, particularly the visual cortex. Our goal was to interpret the cortical changes associated with the disease and the adaptive processes that follow, as revealed by various fMRI methods.

### Disease-related changes

**Figure 6:**
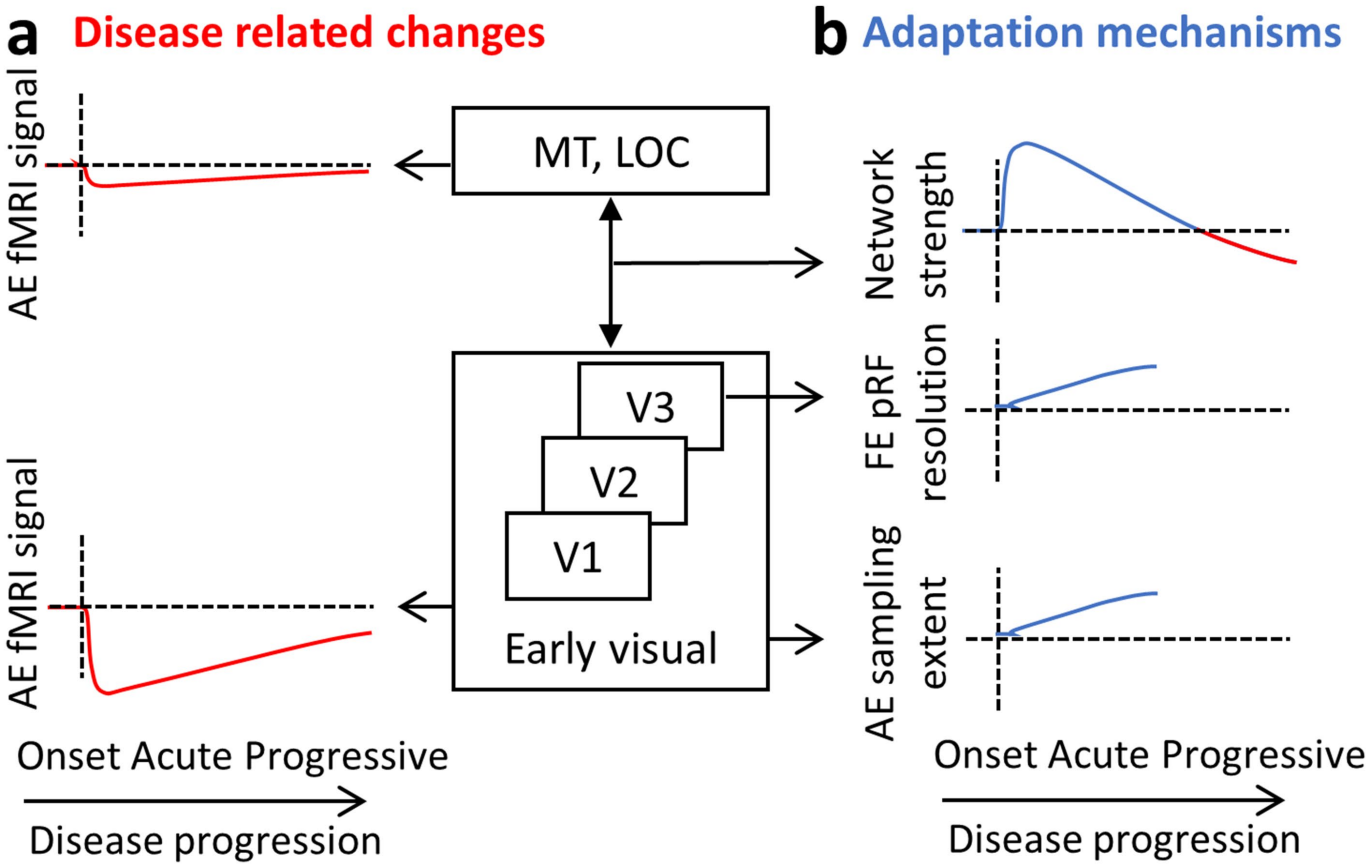
ON results summary. A diagram of the visual hierarchy and sketches representing the results obtained by the different fMRI methods. Results are suggested to reflect the **(a)** disease-related changes on the one hand (in red) and **(b)** the adaptation mechanisms on the other (in blue). AE, affected eye; FE, fellow eye.

During the acute phase following the onset of optic neuritis, functional MRI reveals a marked decrease in the BOLD signal from the affected eye, especially within early visual areas. We believe this reduction in activity reflects a disruption of visual input to the cortex (Figure 6a) and occurs in parallel with the visual symptoms experienced by the patient, such as diminished acuity and contrast sensitivity. We interpret this signal reduction as a direct manifestation of altered sensory input rather than a change in cortical processing per se. Since optic neuritis predominantly impairs visual acuity, rather than higher-order aspects of visual perception, the impact is most evident in early-stage visual regions (e.g., V1 and V2), rather than in higher-level visual cortical areas responsible for more abstract or integrative functions. Indeed, in normally sighted individuals, high-level visual areas such as the LOC respond robustly to shape stimuli and are largely invariant to contrast levels (50). In other words, contrast has little effect on LOC activation, while early visual areas such as V1 show steep, contrast-dependent BOLD responses and are therefore more sensitive to changes in visual acuity.

In addition to this localized activity drop, a general reduction in the functional strength of the visual network is observed over the course of years. We believe this reflects a more systemic impact of the disease, especially in the context of multiple sclerosis (MS), where demyelination affects distributed regions of the brain. This general connectivity reduction, not specific to the visual network, tends to be more pronounced in patients with progressive MS (51, 52). Those progressive patients, both with and without ON, show longer VEP latencies and thinner retinal nerve fiber layer (RNFL) (36). In patients with ON, however, suggested adaptive mechanisms such as increased visual network connectivity may counteract this reduction to some extent in the first few months. As a result, significant decreases in visual network connectivity are typically only apparent in ON patients who also have progressive MS, where the adaptive capacity may be overwhelmed by ongoing neurodegeneration (Figure 6b; upper panel; red line).

Similar disruptions have been observed in glaucoma, where functional imaging has consistently shown cortical consequences of visual field loss. For example, block-design fMRI studies have revealed reduced activation in regions of the visual cortex corresponding to areas of vision loss (53). Additionally, resting-state fMRI (rs-fMRI) has demonstrated impaired functional connectivity, further indicating disruption to large-scale neural networks (54, 55). These findings support the view that both ON and glaucoma lead not only to local visual deficits but also to changes in the organization and communication of broader cortical networks.

### Adaptation mechanisms

Evidence from rs-fMRI suggests that during the acute phase following ON, the visual network shows signs of enhanced functional connectivity. (Figure 6b; upper panel; blue line). This increased connectivity within the visual network is likely an adaptive response to the disruption in visual input and is not seen in MS patients without ON. Therefore, it may represent a specific mechanism aimed at compensating for the loss of signal from the affected eye. One plausible interpretation is that the brain attempts to integrate weaker or more variable input from the affected eye with the more stable input from the fellow eye, enhancing binocular cooperation. This compensatory increase in connectivity may help stabilize visual perception during the early stages of recovery and set the stage for longer-term structural and functional reorganization.

Beyond these network-level adjustments, longer-term adaptation involves specific changes to the properties of neuronal populations in early visual areas. One such change is observed in area V3, where the pRF size associated with the fellow eye becomes smaller. This reduction may serve to preserve or even enhance spatial resolution in the presence of degraded input from the affected eye (Figure 6b; middle panel). Under typical conditions, receptive field sizes increase progressively along the visual hierarchy, from V1 to higher-order areas, allowing for broader integration of visual information and a shift from fine detail to a more generalized perception of scene content. However, in the case of ON, maintaining higher spatial resolution may be more beneficial, and this could be achieved by constraining the usual growth in pRF size. By doing so, the visual system may preserve the reliability of signals processed through the fellow eye, partially offsetting the degraded input from the affected eye. This corresponds to electrophysiological data showing delayed latencies in the fellow eye, which is suggested to reflect adaptive mechanisms at the cortical level that improve binocular integration over time to adjust for the damage incurred. Receptive field remapping, particularly shifts in receptive field location, has also been documented in patients with glaucoma, reinforcing the idea that such changes are part of a broader adaptive response to visual disruption (53).

A second, complementary adaptation is seen in the neuronal populations that process input from the affected eye. In these populations, the connective field size increases (Figure 6b; lower panel). This likely reflects a compensatory strategy aimed at integrating a larger pool of noisy or unreliable inputs. Conceptually, this is akin to zooming out from a blurry image: by pooling information across a wider area, the brain may be able to extract a more coherent signal from degraded input. This form of spatial integration could help maintain functional output even in the presence of reduced input quality.

Interestingly, a contrasting pattern has been reported in glaucoma patients, where connective field mapping revealed smaller connective fields in the visual cortex compared to controls (56). The authors had initially hypothesized that adaptive mechanisms would lead to connective field enlargement, similar to what is observed in ON. However, since the opposite was found, they suggested alternative explanations, potentially involving structural degeneration. This difference may also reflect the distinct disease mechanisms of ON and glaucoma. In glaucoma, a chronic and progressive condition, ganglion cell loss leads to ongoing and irreversible deterioration of vision. By contrast, in ON, the visual deficit is more dynamic: improvement may occur through remyelination, and ganglion cell loss is not present at first (57). This discrepancy underscores the need for further investigation into the specific conditions under which different forms of visual cortex adaptation occur.

### Future directions

In ON, brain activity measured by fMRI correlates closely with ophthalmologic indicators of disease severity, including visual perimetry, contrast sensitivity and visual acuity ((8, 18, 19); for review, see (58)). These associations are observed across multiple visual regions, such as the lateral geniculate nucleus (LGN), early visual cortex, lateral occipital complex (LOC) and extra-striate cortex, and they persist even after controlling for the extent of optic nerve damage, suggesting genuine cortical reorganization. Together, these findings underscore the tight coupling between optic nerve integrity, cortical activation and visual performance in ON. Integrating such measures in longitudinal studies may help identify neural biomarkers predictive of visual recovery, guide targeted therapeutic interventions and stratify patients according to their risk of persistent visual deficits.

### fMRI limitations

Despite its value in understanding neural processes, fMRI has several limitations when applied to patients. One major drawback is the significant time required for both data acquisition and analysis. Scanning sessions can be lengthy, which may be challenging for patients with medical conditions, and the complex data processing demands highly qualified researchers. Additionally, advanced fMRI sequences, such as those used for population receptive field or connective field mapping, are not widely available in standard clinical settings, limiting accessibility. Even when fMRI results provide detailed insights into cortical function, their direct clinical utility remains limited; they rarely influence immediate diagnosis or treatment decisions. Other challenges include the high costs associated with fMRI scans, motion artifacts that can degrade data quality (especially in elderly or visually impaired patients), and the need for specialized software and expertise to interpret results accurately. These factors collectively make fMRI a powerful but impractical tool for routine clinical use.

## Conclusion

Functional MRI has provided valuable insights into optic neuritis (ON), showing how the brain reflects and adapts to visual disruptions through changes in functional connectivity, receptive field properties and network organization. Techniques such as block-design fMRI, resting-state fMRI and population receptive field mapping reveal the brain’s plasticity in response to sensory loss. Despite its strengths, fMRI faces limitations, including long scanning times, complex data analysis and limited clinical application. However, new analytical methods such as graph theory and temporal receptive field mapping continue to expand its potential. Further research is needed to refine these techniques and translate fMRI findings into clinical practice, ultimately advancing our understanding of visual disorders and improving diagnosis and treatment.
